# Use of the All Patient Refined-Diagnosis Related Group (APR-DRG) Risk of Mortality Score as a Severity Adjustor in the Medical ICU

**DOI:** 10.4137/ccrpm.s544

**Published:** 2008-04-18

**Authors:** Daniel Baram, Feroza Daroowalla, Ruel Garcia, Guangxiang Zhang, John J. Chen, Erin Healy, Syed Ali Riaz, Paul Richman

**Affiliations:** 1 Division of Pulmonary/Critical Care Medicine; 2 Department of Preventive Medicine, Stony Brook University School of Medicine, Stony Brook, NY 11794; 3 Center for Public Health and Health Policy Research, Stony Brook University Medical Center; 4 Department of Medicine, Methodist Hospital, Brooklyn NY

**Keywords:** intensive care unit, hospital mortality, benchmarking, severity of illness scoring, risk adjustment

## Abstract

**Objective:**

To evaluate the performance of APR-DRG (All Patient Refined—Diagnosis Related Group) Risk of Mortality (ROM) score as a mortality risk adjustor in the intensive care unit (ICU).

**Design:**

Retrospective analysis of hospital mortality.

**Setting:**

Medical ICU in a university hospital located in metropolitan New York.

**Patients:**

1213 patients admitted between February 2004 and March 2006.

**Main results:**

Mortality rate correlated significantly with increasing APR-DRG ROM scores (p < 0.0001). Multiple logistic regression analysis demonstrated that, after adjusting for patient age and disease group, APR-DRG ROM was significantly associated with mortality risk in patients, with a one unit increase in APR-DRG ROM associated with a 3-fold increase in mortality.

**Conclusions:**

APR-DRG ROM correlates closely with ICU mortality. Already available for many hospitalized patients around the world, it may provide a readily available means for severity-adjustment when physiologic scoring is not available.

Mortality rates vary widely across intensive care units (ICU) in large part attributable to variation in patient acuity and co-morbidities. Severity adjustment of outcomes data is required to track an ICU’s performance over time. For instance, if an ICU institutes a systematic change in practice and the mortality rate is unchanged, it is possible that the change in practice was effective but that the ICU severity also increased hiding the actual improvement in outcome. Also comparing an ICU’s outcome or cost utilization to another ICU or to an external benchmark requires a measure of its severity of illness.

Various ICU severity scores are in use. The Acute Physiology and Chronic Health Evaluation (APACHE) score first developed in 1981 combined physiologic status at 24 hours after ICU admission, demographic information, primary ICU diagnosis and medical co-morbidities to determine severity of illness. After several modifications over the years, APACHE IV can calculate predicted mortality rates and ICU length of stay (Knaus et al. 1981; Knaus et al. 1985; Zimmerman et al. 2006). Simplified Acute Physiology Score (SAPS) II utilizes 17 variables collected after 24 hours in the ICU. (Le Gall et al. 1993) It differs from APACHE in that it is not adjusted for primary ICU diagnosis. Mortality Probability Models (MPM) utilizes binary entry of 11 variables and is less physiologically based than APACHE or SAPS.(Lemeshow et al. 1985) It can be calculated at 0, 24, or 48 hours after admission and returns a predicted mortality rate. Specialized scoring systems have been developed for specific ICU populations, for example trauma, pediatrics and cardiac surgery (Boyd et al. 1987; Balakrishnan et al. 1992; Tu et al. 1994).

Physiologic severity scoring requires dedicated personnel with specialized training to extract vital signs, laboratory values and determine the primary ICU admission diagnosis. Overall accuracy of APACHE scoring is high, but errors do occur (Polderman et al. 2001; Goldhill and Sumner, 1998; Arts et al. 2003; Gooder et al. 1997). Utilizing personnel with years of clinical ICU experience improves the quality and accuracy of the data extraction. Reviews of severity adjustment have been published and the accuracy of various scoring systems accuracy has been directly compared. (Herridge, 2003; Castella et al. 1995).

Though severity scoring has been available for over 30 years, most ICUs in the United States do not routinely calculate severity adjusted outcomes. This is likely due to the financial cost and logistical burden of collecting physiologic data at the bedside. Typically one specially trained full-time employee per ICU is required to collect daily bedside information for each admission. A fully integrated system that includes an electronic medical record and a physiologic scoring system would minimize this cost, but few hospitals have access to an electronic medical record.

The All Patient Refined-Diagnosis Related Group (APR-DRG) methodology was developed by 3M to allow analysis of outcomes across large cohorts for a given diagnostic group (Iezzoni et al. 1995). The APR-DRG scores are calculated from discharge billing codes and are based on primary and secondary discharge diagnosis, age, and preexisting medical conditions. In addition to other scores, APR-DRG ranks the risk of mortality (ROM) as low, medium, high, and extreme. This proprietary scoring system specifically excludes codes reflecting in-hospital complications. There is a paucity of data regarding the APR-DRG methodology in the ICU setting, and the accuracy of APR-DRG ROM for severity adjustment of ICU patients has not been reported.

In this paper we evaluated the performance of APR-DRG ROM as a mortality risk adjustor in the ICU.

## Methods

### Setting

Stony Brook University Medical Center’s Medical ICU is a 12 bed ICU in a 504 bed tertiary-care university hospital located in the metropolitan New York area. It is a closed ICU managed by boardcertified pulmonary/critical care attendings who supervise medical housestaff and pulmonary/critical care fellows.

### Data

1213 patients admitted to the medical ICU between February 1, 2004 and March 31, 2006 were identified. Demographic and hospital discharge status were obtained.

APR-DRG ROM (3M, Version 20) was available through the University Hospital Consortium, as Stony Brook University Medical Center is a member of this organization. APR-DRG calculations are based on electronic bills submitted by the hospital at time of patient discharge after chart data extraction by certified professional coders. Primary DRG billing diagnosis was identified and divided into 5 groups: cardiology, gastroenterology, neurology, pulmonary, and other.

Though ICU severity adjustment was not performed routinely in our ICU, APACHE II scores were available for 165 patients admitted from April 2004 through August 2004, and APACHE III scores were available for 204 patients admitted from April 2005 through August 2005 and from December 2005 through January 2006. APACHE II and APACHE III scores were performed by dedicated personnel. Scores were calculated 24 hours post-ICU admission based on information in the medical record using standardized computerized spreadsheets.

All data were fully de-identified and the Stony Brook University Institutional Review Board approved the project.

### Statistical analysis

The demographic information was summarized by descriptive statistics (means and standard deviations for continuous variables, and frequencies and percentages for categorical variables). Disease group-specific mortality rates for different APR-DRG ROM score levels were also calculated. Using ROM “Low” as the reference group, in-hospital mortality odds ratios (OR) and their 95% confidence intervals (CI) for other ROM categories (“Moderate”, “High”, and “Extreme”) were calculated. The Cochran-Armitage test (Agresti, 2002) was used to evaluate the linear trend between ROM scores and mortality rates. With ROM score as a continuous predictor, we obtained the average OR (95% CI) using logistic regression analysis. Area under the receiver operating characteristics (ROC) curve (AUC) was also calculated.

Multivariable logistic models were developed to further evaluate the mortality predictive power of APR-DRG ROM, while adjusting for other covariates, including age, gender, and disease groups. The possible 2-way interaction terms among these four variables were also considered in the model building process using the likelihood ratio test (LRT). The model discrimination and calibration were evaluated by the AUC and Hosmer-Lemeshow goodness-of-fit (GOF) test, respectively. The performances of candidate models were evaluated using 5-fold cross-validation (Zhang, 1993). Data from all patients were first randomly grouped into 5 subsets of equal size. Each of the 5 subsets was in turn treated as a validation set with the remaining 4 subsets as the training set. Thus, there were five pairs of training sets and testing sets. The regression coefficient estimates based on the current training set were used to calculate the predicted probabilities of death for the current testing set. These predicted mortality rates were used to compute the AUC and Hosmer-Lemeshow goodness-of-fit (GOF) test p-value for the current testing set(Hosmer, 2000). The five validated AUC values were used to produce each model’s cross-validated statistics and its standard deviation. AUC values between (0.70, 0.80) were considered acceptable discrimination; while AUC > 0.80 was considered excellent discrimination (Hosmer and Lemeshow, 2000). All statistical analyses were performed in SAS 9.1 and a two-sided p-value of less than 0.05 was treated as statistically significant.

## Results

APR-DRG ROM scores of 1213 patients were included in the analysis. Of these, 165 had APACHE II scores (range 2–43, with a mean of 20.7 and a standard deviation of 7.8) and 204 had APACHE III scores (range 8–209, with a mean of 65.7 and a standard deviation of 30.6). Of the 1213 patients, the mean (SD) age was 62.2 years (18.3 years) and 46.6% (n = 565) were females.

**Figure 1.**
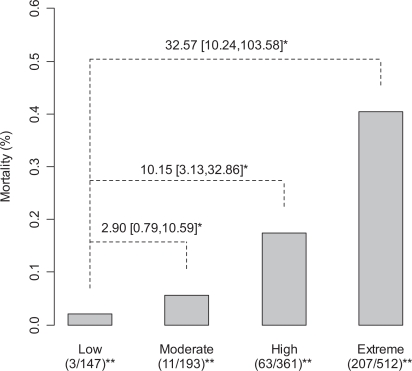
Comparison of mortality rates among different APR-DRG ROM scoring categories. **Notes:** *Mortality odds ratios and their 95% confidence intervals for ROM categories (“Moderate”, “High”, and “Extreme”), using ROM “Low” category as the reference group. **Number of deaths and number of subjects for each ROM category included in the analysis.

The association of mortality and APR-DRG ROM scores was consistent within various disease groups, i.e. higher APR-DRG ROM scores tended to be associated with increased mortality (Table 1). The crude odds ratios of two higher levels (“High” and “Extreme”) of APR-DRG ROM scores are statistically significantly higher than the “Low” ROM score (OR for “High vs. Low”= 10.15 [95% CI: 3.13,32.86]; OR for “Extreme vs. Low” = 32.57 [95% CI: 10.24,103.58]), although the difference between level “Moderate” and level “Low” was less dramatic (OR for “Moderate vs. Low” = 2.90 [95% CI: 0.79,10.59]) (Fig. 1). The linear trend between ROM scores and in-hospital mortality rates was highly significant based on the Cochran-Armitage test (p < 0.0001). When the four levels of APR-DRG ROM scores were treated as a continuous predictor in a simple logistic regression model, one unit of increase in APR-DRG ROM was associated with an average 3.26-fold increase in mortality rate, with a 95% confidence interval of (2.64, 4.03) (p-value < 0.0001). The associated AUC was 0.733 for APR-DRG ROM, an acceptable discrimination.

To adjust for other relevant covariates, i.e. age, gender and disease group, multivariable logistic models were developed. Possible 2-way interaction terms among the above four predictors were not statistically significant in the model building process based on LRTs. Neither was the main effect of gender. Therefore, there were 4 candidate models to be considered. Performance of these candidate models, based on five-fold cross-validation analysis and model selection results, are summarized in Table 2.

The overall best model, in terms of LRT, discrimination (average cross-validation AUC for testing set) and calibration (the Hosmer-Lemeshow goodness of fit p-value for testing set), was Model D with APR-DRG ROM, age, and disease groups as predictors. All three predictors in Model D were significant based on LRT, suggesting that APR-DRG ROM, age, and disease groups were all significantly and independently associated with mortality risk in patients. The five-fold cross-validation results from Model D showed an average AUC of 0.762 with the smallest standard deviation of 0.021, Based on all available data, the adjusted odd ratio (95% CI) of APR-DRG ROM score for Model D was 3.01 (2.40, 3.76), and the AUC value was 0.782.

## Discussion

The APR-DRG ROM performed very well in grouping our ICU patients with significantly different mortality rates and provided acceptable discrimination in regards to mortality. This suggests APR-DRG ROM may be a useful means for severity adjustment in the ICU.

The AUC for APR-DRG ROM was 0.733. In a large international study with over 14,000 patients, the AUC for APACHE, MPM, and SAPS, AUC ranged from 0.766–0.861(Castella et al. 1995); newer scoring systems were shown to have improved discrimination than older. In our study, based on relatively small subset of patients who had APACHE scoring, APACHE II and APACHE III provided either similar or slightly more discriminative power than APR-DRG ROM in regards to mortality rate, with AUC = 0.768 for APACHE II (n = 165) and AUC = 0.829 for APACHE III (n = 204). That APACHE was better calibrated than APR-DRG ROM is not surprising as it was specifically designed for critically ill patients, whereas APR-DRG was calibrated for patients across a wider spectrum of acuity. Addition of age and disease group increased the APR DRG ROM AUC up to 0.782; perhaps future calibration with larger datasets would increase the AUC up into the range of the more established scoring systems.

APR-DRG ROM differs from APACHE and most other ICU severity scoring methodologies in several important ways. First APR-DRG ROM is calculated after hospital discharge; other scoring systems are calculated within 24 or 48 hours of ICU admission. Therefore the APR-DRG ROM cannot be calculated at time of ICU admission and cannot be used by the clinician caring for the patient. MPM and APACHE IV allow for repeat assessments which may provide the bedside practitioner an assessment of patient status; APR DRG ROM can not provide this.

Second, APR-DRG ROM was designed to correlate with survival to hospital discharge, not ICU mortality. Earlier scoring systems predicted ICU mortality, such that patients who were discharged from the ICU but died prior to hospital discharge were considered survivors. As many patients destined to die are transitioned to non-ICU care at the end of their hospital course, the distinction between ICU and hospital mortality becomes blurred.

Lastly, the major difference between APR-DRG ROM and other ICU severity scoring systems is that calculation of APR-DRG ROM does not require dedicated personnel and resources for ICU data collection. Scoring is calculated using coding information available for all U.S. hospital discharges. At present 1500 hospitals utilize the APR-DRG methodology, and therefore APR-DRG ROM scores are already currently being calculated for a large number of ICU patients. These scores could be used to severity adjust ICU outcomes, however they are not being reported back to the ICU. All other ICU scores require entry of physiologic status; this requires dedicated personnel for data extraction from the bedside record.

Professional coders extract chart data for purposes of billing, reimbursement, and quality assurance initiatives. Though coding accuracy is dependent on the expertise of the coder, tightly regulated coding laws and credentialing serve to limit variation in practice and accuracy (Campbell et al. 2001). Hospitals have an interest in accurate coding as it is the primary determinant of reimbursement and there are significant financial and operational penalties for improper coding. Though the DRG coding system was initially designed in the United States by the Social Security Administration for use in Medicare reimbursement (Scott, 1984), it has become increasingly used in Europe and elsewhere. Per the 3M corporate website, the APR-DRG methodology is being used in over 26 countries worldwide.

The APR-DRG is marketed to hospital as a means to benchmark their overall performance and resource utilization. It allows comparison of how a given institution is performing for a selected DRG, such as its performance in caring for patients with pneumonia. Several states require utilization of the APR-DRG methodology for all hospital discharges in order to allow comparison between hospitals. Despite its widespread use, there is little in the peer-reviewed literature regarding APR-DRG and there are no papers describing its validity or accuracy in the ICU setting.

There are several limitations in the analysis. Our data only include patients in a single medical ICU; APR-DRG ROM may perform differently in different patient populations such those caring for surgical or pediatric patients. In certain subgroups, clinical issues are often a prime determinant of outcome, such as pre-operative left ventricular function in cardiac surgery patients. As the APR-DRG methodology does not factor clinical variables, it may not perform as well as dedicated scoring systems for these patients.

Use of the APR-DRG ROM score as a severity of illness adjustor in the ICU requires validation with much larger populations and across hospitals. Studies comparing other methodologies have shown significant variation across hospitals (Knaus et al. 1993; Sirio et al. 1999; Render et al. 2005). Questions remain how much of this variation is from true differences in outcome and how much is dependant on the scoring system utilized (Puri, 2005; Capuzzo et al. 2000; McNelis et al. 2001; Beck et al. 2003; Schafer et al. 1990; Iezzoni et al. 1995).

Despite reservations regarding severity adjustment, there are growing calls to benchmark ICU outcomes and to report severity adjusted mortality (Garland, 2005; Sirio, 2006; Combes et al. 2005; Gupta et al. 2002), and organizations such as The Joint Commission on Accreditation of Healthcare Organizations (JCAHO) and the Leapfrog Group are interested in standardizing ICU severity adjustment (Afessa et al. 2005). With the increasing pressure to standardize ICU protocols and care, it is important that we accurately measure severity-adjusted outcomes to ensure that these protocols are not improving one aspect of care at the cost of another.

## Conclusions

APR-DRG ROM correlates closely with ICU mortality. As this score is already available in many hospitals around the world, it provides a readily available means for severity-adjusting ICU outcomes when physiologic scoring is not available.

## Figures and Tables

**Table 1.** Summary of mortality rates for different APR-DRG Risk of Mortality (ROM) levels and five disease groups.

**Disease Group (n)**	**ROM = Low**			**ROM = Moderate**			**ROM = High**			**ROM = Extreme**		
	dead	total	mortality %	dead	total	mortality %	dead	total	mortality %	dead	total	mortality %
Cardiology (113)	0	2	0.0	0	14	0.0	7	45	15.6	22	52	42.3
Gastroenterology (172)	0	33	0.0	0	38	0.0	6	52	11.5	17	49	34.7
Neurology (94)	0	8	0.0	3	14	21.4	7	19	36.8	34	53	64.2
Pulmonary (261)	2	32	6.3	4	46	8.7	16	101	15.8	36	82	43.9
Other (573)	1	72	1.4	4	81	4.9	27	144	18.8	98	276	35.5
Total (1213)	3	147	2.0	11	193	5.7	63	361	17.5	207	512	40.4

**Table 2.** Summary of 5-fold cross-validation and model selection results for four candidate models.

**Model**	**Training Set**			**Testing Set**			**All Data**				
	AUC*	**Mean (SD)**	**AUC**	**Mean (SD)**	GOF** p-value	**AUC**	**GOF p-value**	**Likelihood 2LogLik**	**Ratio Test p-value**	**OR for ROM (95% CI)**
A	0.723	0.733	0.771	0.734	0.40	0.733	0.98	1138.851	NA	3.26
	0.736	(0.008)	0.724	(0.029)	0.30					(2.64,4.03)
	0.740		0.707		0.27					
	0.740		0.710		0.047					
	0.727		0.758		0.29					
B	0.758	0.771	0.818	0.771	0.13	0.770	0.77	1105.156	<0.0001†	3.06
	0.769	(0.008)	0.779	(0.031)	0.71					(2.45,3.81)
	0.777		0.741		0.48					
	0.778		0.743		0.26					
	0.771		0.772		0.071					
C	0.758	0.759	0.715	0.730	0.68	0.758	0.82	1114.064	<0.0001†	3.24
	0.755	(0.005)	0.719	(0.027)	0.053					(2.61,4.02)
	0.757		0.732		0.04					
	0.767		0.708		0.24					
	0.756		0.776		0.82					
D	0.779	0.783	0.740	0.762	0.15	0.782	0.36	1081.930	0.0001††	3.01
	0.780	(0.004)	0.779	(0.021)	0.53					(2.40,3.76)
	0.783			0.762		0.59					
	0.790			0.743		0.77					
	0.782			0.787		0.12					

**Notes:** Independent variables used in the logistic model: Model A: ROM. Model B: ROM + Age. Model C: ROM + Disease Groups. Model D: ROM + Age + Disease Groups.

* Area under ROC.

** Hosmer-Lemeshow Goodness of Fit.

† Likelihood Ratio Test Relative to Model A.

†† Likelihood Ratio Test Relative to Model B.
